# Supplementary material for: The PRC2.1 Subcomplex Opposes G1 Progression through Regulation of CCND1 and CCND2
Source: bioRxiv. 2024 Oct 16:2024.03.18.585604. Originally published 2024 Mar 19. Preprint. [Version 2] doi: 10.1101/2024.03.18.585604 (PMC10983909; doi:10.1101/2024.03.18.585604)

bioRxiv preprint doi: https://doi.org/10.1101/2024.03.18.585604; this version posted October 16, 2024. The copyright holder for this preprint (which was not certified by peer review) is the author/funder, who has granted bioRxiv a license to display the preprint in perpetuity. It is made available under aCC-BY 4.0 International license.

Supplemental Figure Legends

Supplementary Figure 1: Dosing to Determine Inhibitor Concentration for Chemogenetic Screen
(S1A): Drug dosing experiments were performed to determine screening concentrations. Cells were counted during passage in increasing doses of camptothecin (*left*), palbociclib (*center*) and colchicine (*right*).
(S1B): Representative images of flow cytometry traces from untreated cells or cells treated with 0.7µM palbociclib, 9.2nM colchicine or 1nM camptothecin treated cells for three days, then stained propidium iodide. Plots represent the number of stained cells with a given propidium iodide intensity.
(S1C): Venn diagrams showing overlap for significant genes that sensitized (*left*) or de-sensitized cells (*right*) to each condition tested. Genes that were determined as significant in all three screens were omitted in further analyses.
(S1D): Dose-response curve of palbociclib-induced proliferation rescue in combination with oxidative phosphorylation inhibitors by PrestoBlue assay. Data represents mean of three technical replicates, normalized to the initial dose of each inhibitor in indicated concentration of rotenone, ±StdDev.
(S1E): Same as in (S1D) but for TTFA.
(S1F): Same as in (S1D) but for Oligomycin.

bioRxiv preprint doi: https://doi.org/10.1101/2024.03.18.585604; this version posted October 16, 2024. The copyright holder for this preprint (which was not certified by peer review) is the author/funder, who has granted bioRxiv a license to display the preprint in perpetuity. It is made available under aCC-BY 4.0 International license.

Supplementary Figure 2: Assays to Determine Resistance of PRC2 Component Mutants to CDK4/6 Inhibitors

(S2A): Schematic of internally controlled competitive proliferation assay used to validate chemogenetic results or knockout cell line proliferation when treated with palbociclib. In experiments where we generated pooled knockouts, GFP^+ cells expressing Cas9 were mixed with GFP^- cells without Cas9 (as in Figure 4E). For competitive proliferation experiments with monoclonal knockout cell lines, GFP^+, Cas9 expressing cells were mixed with GFP^- monoclonal knockout lines (as in Supplemental Figure 2C).

(S2B): Western blots demonstrating the efficacy of indicated sgRNA used in the competitive proliferation assay.

(S2C): Competitive proliferation assay for or monoclonal knockout cell lines. wild-type, MTF2Δ and JARID2Δ cell lines (GFP^-) were mixed with wild-type cells expressing Cas9 and GFP (GFP^+) and treated with either DMSO (mock) or 1.5μM palbociclib (*left*), 3.5μM ribociclib (*center*) or 0.4μM abemaciclib (*right*). Cells were split every three days and the GFP^-/GFP^+ ratio was assessed every six days by flow cytometry.

(S2D): Western blot of protein extracts from cells treated with DMSO (mock) or 1.5μM palbociclib for 48 hours, probed with indicated antibody. PARP cleavage and BIM from protein extracts from RPE1 cells over-expressing a doxycycline-inducible HA-tagged BIM to induce apoptosis as a control.

Supplementary Figure 3: Analysis of Changes in H3K27me3 Distribution in CUT&RUN and Differentially Expressed Genes in RNA-Seq Experiments

(S3A): Venn diagrams of the Gencode Annotations of promoters that had significantly up regulated (top row) and down regulated H3K27me3 (bottom row) for MTF2Δ (*left*) and JARID2Δ cells (*right*). Significant promoters were determined as having a $\log_2$ fold change ±1 and an adjusted p-value of <0.1.

(S3B): Same as in (S3B) only for our RNA-Seq experiments and significant promoters were determined as having a $\log_2$ fold change ±1 and an adjusted p-value < 0.05.

(S3C): *Top* - PCA plot of H3K27me3 peaks called by macs2 from CUT&RUN experiment done in biological duplicate. *Bottom* – PCA plot of RNA-seq reads for experiment in biological triplicate.

(S3D): Average H3K27me3 distribution over a 10kb window for 1,877 peaks overlapping with CGIs. Genomic regions are ordered by the H3K27me3 read density intensity in wild-type cells then plotted for the same loci in MTF2Δ and JARID2Δ cells. Plots are of one of two biological replicate.

(S3E): Bar plot of $\log_{10}$(p-value) of Reactome (teal bars) and MSigDB (red bars) terms associated with promoters of protein coding genes that contain at least one CGI.

(S3F): Bar plot of -$\log_{10}$(p-value) for the enrichment of a given transcription factors from ENCODE and ChEA databases binding to the list of promoters with overlapping GGIs and H3K27me3 peaks.

Supplementary Figure 4: Analysis of Differential H3K27me3 Distribution and Transcript Expression of D-type Cyclins in CUT&RUN and RNA-Seq Data Sets

(S4A): Volcano plot of DESeq2 calculated changes in $\log_2$ fold-change in H3K27me3 signal in promoters versus the $\log_{10}$(p-value) in enrichment in MTF2Δ cells determined by CUT&RUN. CCND1 and CCND2 location within the dataset are indicated by yellow dots.

(S4B): Same as in (S4A) but for transcript abundance determined by RNA-seq of MTF2Δ cells.

(S4C): Volcano plot of DESeq2 calculated changes in $\log_2$ fold-change in H3K27me3 signal in promoters versus the $\log_{10}$(p-value) in enrichment in JARID2Δ cells determined by CUT&RUN. CCND1 and CCND2 location within the dataset are indicated by yellow dots.

(S4D): Same as in (S4C) but for transcript abundance determined by RNA-seq of JARID2Δ cells.

(S4E): Scatter plot of $\log_2$ fold-change in transcript abundance vs H3K27me3 promoter signal for genes with an adjusted p-value <0.1 in our CUT&RUN and adjusted p-value <0.05 in our RNA-Seq from JARID2Δ cell lines.

(S4F): Genome browser traces of H3K27me3, transcript coverage and CGI location within the CCND3 promoter region.

bioRxiv preprint doi: https://doi.org/10.1101/2024.03.18.585604; this version posted October 16, 2024. The copyright holder for this preprint (which was not certified by peer review) is the author/funder, who has granted bioRxiv a license to display the preprint in perpetuity. It is made available under aCC-BY 4.0 International license.

Supplementary Figure 5: Regulation of D-type Cyclin Expression by PRC2.1 and PRC2.2

(S5A): Quantification of protein signal from western blot in Fig. 6C for CCND1 (*left*), CCND2 (*center*), and CCND3 (*right*) normalized to Actin. Each bar is the mean for three biological replicates, error bars ±StDev. *: p-value<0.05, **: p-value<0.005, ***: p-value<0.0005, n.s.: not significant, two tailed unpaired Student's t-test.

(S5B): Western blots of whole-cell lysates of three-independently isolated monoclonal SUZ12Δ, MTF2Δ and JARID2Δ knockout cell lines probed with the indicated antibodies.

(S5C): qRT-PCR relative quantification of CCND1, CCND2 and CCND3 mRNA levels in wild-type, SUZ12Δ, MTF2Δ and JARID2Δ cells, three biological replicates, performed in technical triplicate, ±StDev. *: p-value<0.05, **: p-value<0.005, ***: p-value<0.0005, n.s.: not significant, two tailed unpaired Student's t-test.

(S5D): Dot plot of $\log_2$ fold-change for indicated mRNAs in MTF2Δ and JARID2Δ cells. Established cut-off for significant $\log_2$-fold change indicated by dashed grey line.

(S5E): Western blot for a panel of G1 regulators from lysates of wild-type, SUZ12Δ, MTF2Δ and JARID2Δ cell lines from three knockout cell lines probed with indicated antibodies.

(S5F): Competitive proliferation assay of CCND1 and CCND2 overexpression cell lines resistance to palbociclib. Wild-type, dox-inducible CCND1 and dox-inducible CCND2 polyclonal HAP1 cell lines (GFP^-) were mixed with wild-type cells expressing GFP (GFP^+) and treated with either DMSO (mock) or 1.5µM palbociclib, in the presence or absence of 500ng/mL doxycycline. Mock and palbociclib-containing media, with or without doxycycline was replaced daily. Cells were split and GFP^-/GFP^+ ratio was assessed by flow cytometry every three days. Fitness of each overexpression of each pool was determined by first normalizing the GFP^-/GFP^+ ratio to the minus doxycycline control and then the ratio of GFP-/GFP+ between the mock and palbociclib conditions.

(S5G): Co-immunoprecipitation of 2xFLAG-2xStrep-CDK6 expressed in wild-type, SUZ12Δ, MTF2Δ and JARID2Δ knockout cell lines, probed with the indicated antibodies.

bioRxiv preprint doi: https://doi.org/10.1101/2024.03.18.585604; this version posted October 16, 2024. The copyright holder for this preprint (which was not certified by peer review) is the author/funder, who has granted bioRxiv a license to display the preprint in perpetuity. It is made available under aCC-BY 4.0 International license.

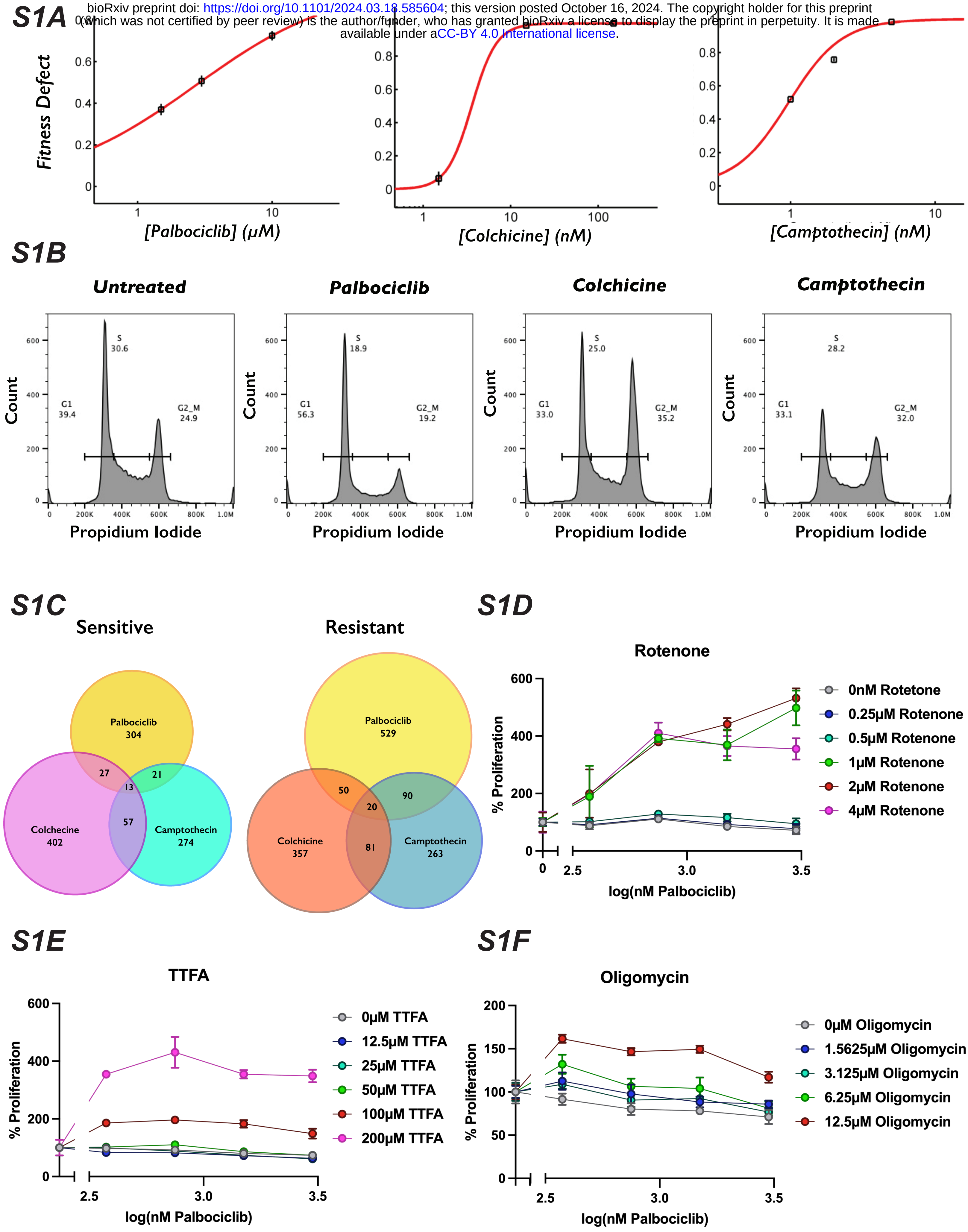

bioRxiv preprint doi: https://doi.org/10.1101/2024.03.18.585604; this version posted October 16, 2024. The copyright holder for this preprint (which was not certified by peer review) is the author/funder, who has granted bioRxiv a license to display the preprint in perpetuity. It is made available under aCC-BY 4.0 International license.

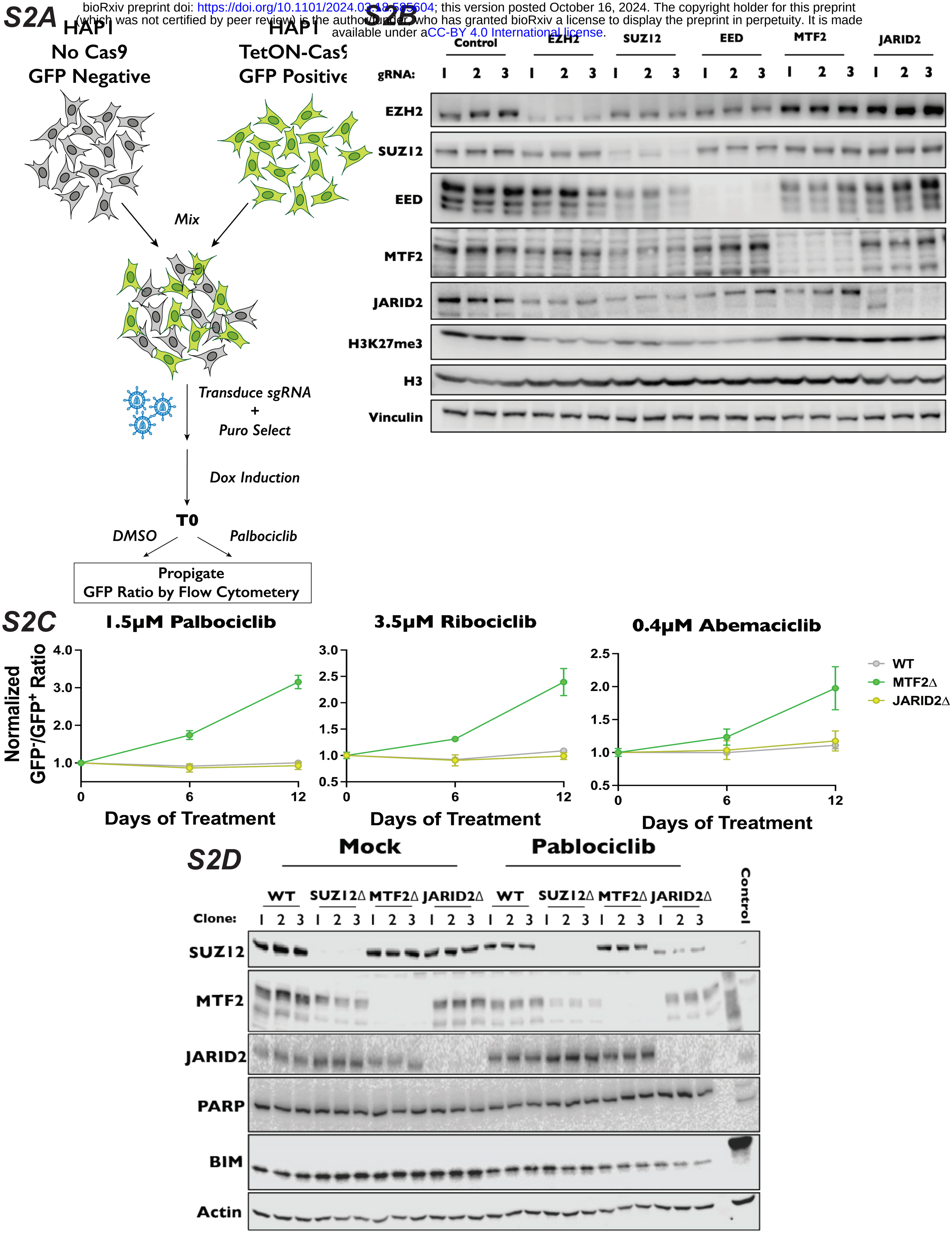

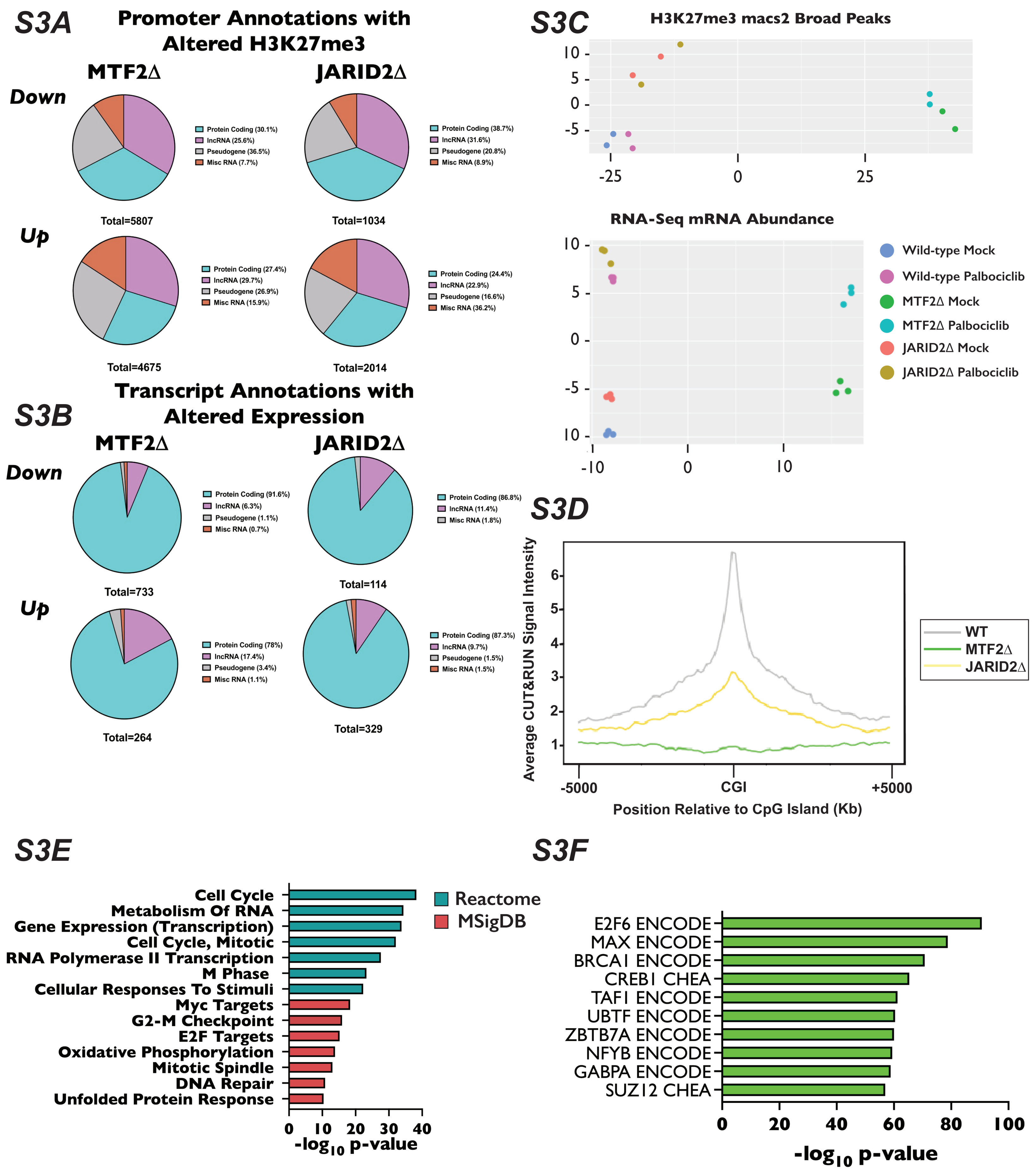
S3A
Promoter Annotations with Altered H3K27me3
MTF2Δ
JARID2Δ
Down
Protein Coding (30.1%)
lncRNA (25.6%)
Pseudogene (36.5%)
Misc RNA (7.7%)
Total=5807
Protein Coding (38.7%)
lncRNA (31.6%)
Pseudogene (20.8%)
Misc RNA (8.9%)
Total=1034
Up
Protein Coding (27.4%)
lncRNA (29.7%)
Pseudogene (26.9%)
Misc RNA (15.9%)
Total=4675
Protein Coding (24.4%)
lncRNA (22.9%)
Pseudogene (16.6%)
Misc RNA (36.2%)
Total=2014
S3B
Transcript Annotations with Altered Expression
MTF2Δ
JARID2Δ
Down
Protein Coding (91.6%)
lncRNA (6.3%)
Pseudogene (1.1%)
Misc RNA (0.7%)
Total=733
Protein Coding (86.8%)
lncRNA (11.4%)
Misc RNA (1.8%)
Total=114
Up
Protein Coding (78%)
lncRNA (17.4%)
Pseudogene (3.4%)
Misc RNA (1.1%)
Total=264
Protein Coding (87.3%)
lncRNA (9.7%)
Pseudogene (1.5%)
Misc RNA (1.5%)
Total=329
S3C
H3K27me3 macs2 Broad Peaks
10
5
0
-5
-25
0
25
RNA-Seq mRNA Abundance
10
5
0
-5
10
-10
0
10
Wild-type Mock
Wild-type Palbociclib
MTF2Δ Mock
MTF2Δ Palbociclib
JARID2Δ Mock
JARID2Δ Palbociclib
S3D
Average CUT&RUN Signal Intensity
1
2
3
4
5
6
WT
MTF2Δ
JARID2Δ
-5000
CGI
+5000
Position Relative to CpG Island (Kb)
S3E
Cell Cycle
Metabolism Of RNA
Gene Expression (Transcription)
Cell Cycle, Mitotic
RNA Polymerase II Transcription
M Phase
Cellular Responses To Stimuli
Myc Targets
G2-M Checkpoint
E2F Targets
Oxidative Phosphorylation
Mitotic Spindle
DNA Repair
Unfolded Protein Response
Reactome
MSigDB
0
10
20
30
40
-log10 p-value
S3F
E2F6 ENCODE
MAX ENCODE
BRCA1 ENCODE
CREB1 CHEA
TAF1 ENCODE
UBTF ENCODE
ZBTB7A ENCODE
NFYB ENCODE
GABPA ENCODE
SUZ12 CHEA
0
20
40
60
80
100
-log10 p-value

bioRxiv preprint doi: https://doi.org/10.1101/2024.03.18.585604; this version posted October 16, 2024. The copyright holder for this preprint (which was not certified by peer review) is the author/funder, who has granted bioRxiv a license to display the preprint in perpetuity. It is made available under aCC-BY 4.0 International license.

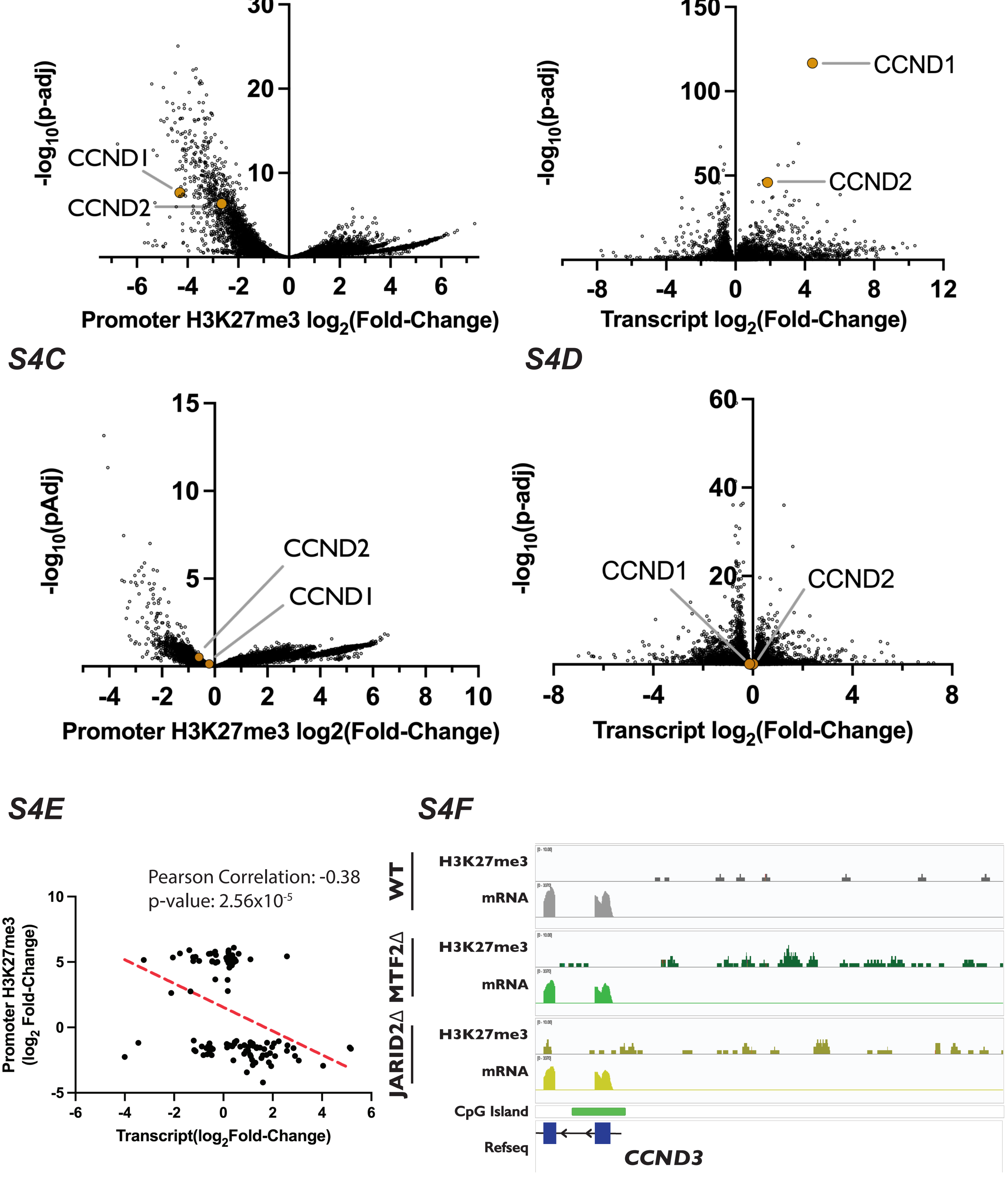

bioRxiv preprint doi: https://doi.org/10.1101/2024.03.18.585604; this version posted October 16, 2024. The copyright holder for this preprint (which was not certified by peer review) is the author/funder, who has granted bioRxiv a license to display the preprint in perpetuity. It is made available under aCC-BY 4.0 International license.

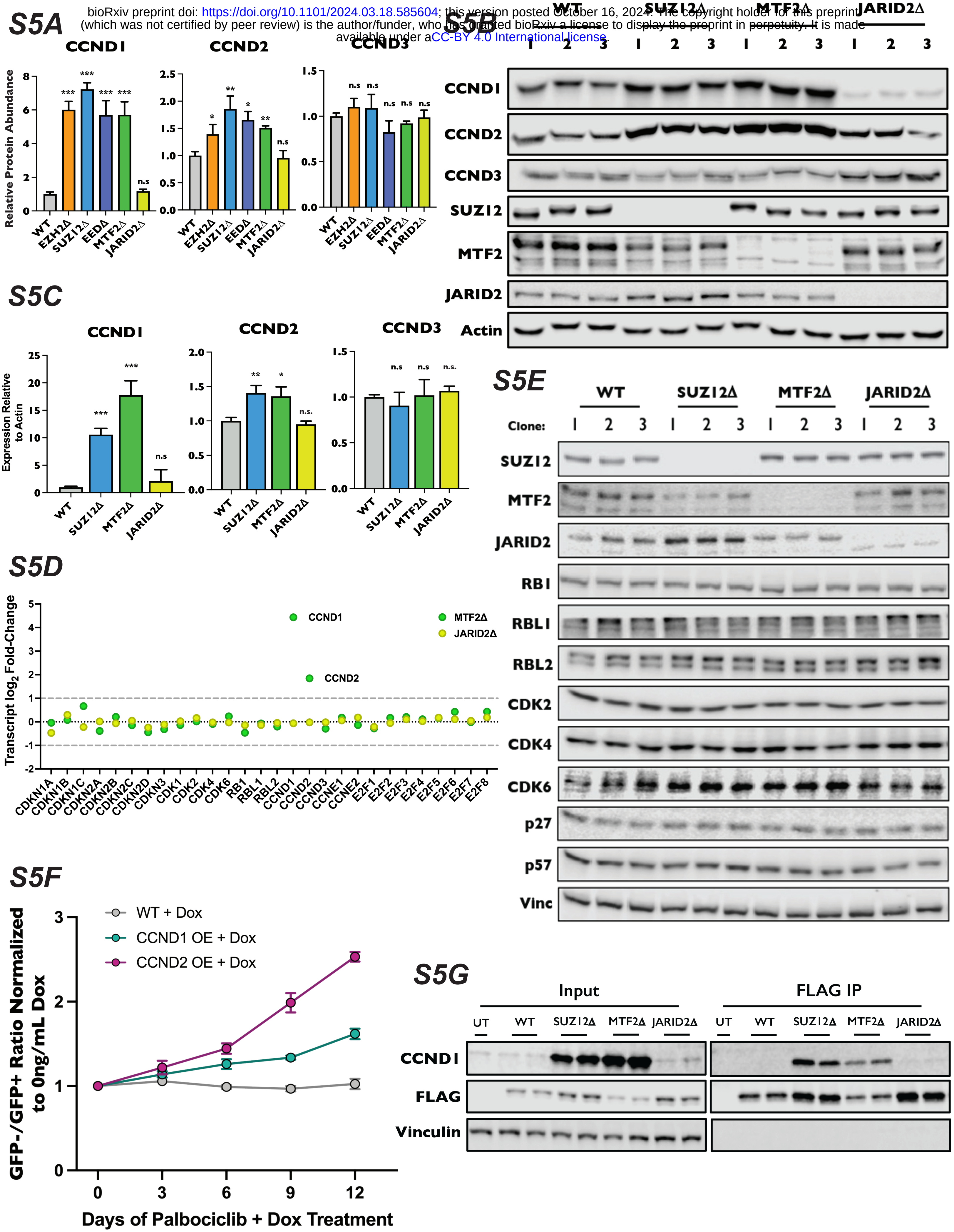

Supplement: Supplement 1 [file NIHPP2024.03.18.585604v2-supplement-1.pdf]
