# Supplementary material for: The PRC2.1 Subcomplex Opposes G1 Progression through Regulation of CCND1 and CCND2
Source: bioRxiv. 2024 Mar 19:2024.03.18.585604. Preprint. [Version 1] doi: 10.1101/2024.03.18.585604 (PMC10983909; doi:10.1101/2024.03.18.585604)

bioRxiv preprint doi: https://doi.org/10.1101/2024.03.18.585604; this version posted March 22, 2024. The copyright holder for this preprint (which was not certified by peer review) is the author/funder, who has granted bioRxiv a license to display the preprint in perpetuity. It is made available under aCC-BY 4.0 International license.

Supplemental Figure Legends

Supplementary Figure 1: Dosing to Determine Inhibitor Concentration for Chemogenomics Screen
(S1A): Drug dosing experiments were performed to determine screening concentrations. Cells were counted during passage in increasing doses of camptothecin (*left*), palbociclib (*center*) and colchicine (*right*).
(S1B): Representative images of flow cytometry traces from untreated cells or cells treated with 0.7μM palbociclib, 9.2nM colchicine or 1nM camptothecin treated cells for three days, then stained propidium iodide. Plots represent the number of stained cells with a given propidium iodide intensity.
(S1C): Venn diagrams showing overlap for significant genes that sensitized (*left*) or de-sensitized cells (*right*) to each condition tested. Genes that were determined as significant in all three screens were omitted in further analyses (see Table S2 for a list of the omitted genes).

Supplementary Figure 2: Assays to Determine Resistance of PRC2 Component Mutants to CDK4/6 Inhibitors
(S2A): Schematic of internally controlled competitive growth assay used to validate chemogenomics results or monoclonal cell line proliferation when treated with palbociclib. In experiments where we generated polyclonal pooled knockouts, GFP^+ cells expressing Cas9 were mixed with GFP^- cells without Cas9 (as in Fig 4E). For competitive growth experiments with monoclonal knockout cell lines, GFP^+, Cas9 expressing cells were mixed with GFP^- monoclonal knockout lines (as in Fig. S2C).
(S2B): Western blots demonstrating the efficacy of indicated sgRNA used in our competitive growth assay and use of polyclonal pooled knockouts. Cells were transduced with the indicated sgRNA, Cas9 expression was then induced for three days with doxycycline to generate polyclonal pooled knockouts. Whole-cell lysates from these pooled knockouts were then probed with antibodies against the indicated proteins.
(S2C): Competitive growth assay for or monoclonal knockout cell lines. Wild-type, MTF2Δ and JARID2Δ cell lines (GFP^-) were mixed with wild-type HAP1 cells expressing Cas9 and GFP (GFP^+) and treated with either DMSO (mock) or 1.5μM palbociclib (*left*), 3.5μM ribociclib (*center*) or 0.4μM abemaciclib (*right*) for twelve days. Cells were split every three days and the GFP^-/GFP^+ ratio was assessed every six days by flow cytometry.
(S2D): Western blot of protein extracts from cells treated with DMSO (mock) or 1.5μM palbociclib for 48 hours, probed with indicated antibody. PARP cleavage and BIM over expression controls are included in the

bioRxiv preprint doi: https://doi.org/10.1101/2024.03.18.585604; this version posted March 22, 2024. The copyright holder for this preprint (which was not certified by peer review) is the author/funder, who has granted bioRxiv a license to display the preprint in perpetuity. It is made available under aCC-BY 4.0 International license.

last lane from protein extracts from RPE1 cells over-expressing a doxycycline-inducible HA-tagged BIM to induce apoptosis.

Supplementary Figure 3: Analysis of Changes in H3K27me3 Distribution in CUT&RUN and Differentially Expressed Genes in RNA-Seq Experiments
(S3A): *Top* - PCA plot of H3K27me3 peaks called by macs2 from CUT&RUN experiment done in biological duplicate. *Bottom* – PCA plot of RNA-seq reads for experiment done in biological triplicate.
(S3B): Venn diagrams of the Gencode Annotations of promoters that had significantly up regulated (top row) and down regulated H3K27me3 (bottom row) from our CUT&RUN experiment for MTF2Δ (*left*) and JARID2Δ cells (*right*). Significant promoters were determined as having a $\log_2$ fold change ±1 and an adjusted p-value of <0.1.
(S3C): Same as in (S3B) only for our RNA-Seq experiments and significant promoters were determined as having a $\log_2$ fold change ±1 and an adjusted p-value < 0.05.
(S3D): Average H3K27me3 distribution over a 10kb window for 1,877 peaks overlapping with CpG islands with the highest average signal intensity were identified in Wild-type cells and plotted for the same loci in MTF2Δ and JARID2Δ cells.
(S3E): Bar plot of $-\log_{10}$(p-value) for the enrichment of a given transcription factors from ENCODE and ChEA databases binding to the list of promoters with overlapping CpG islands and H3K27me3 peaks.

Supplementary Figure 4: Analysis of Differential H3K27me3 Distribution and Transcript Expression of D-type Cyclins in CUT&RUN and RNA-Seq Data Sets
(S4A): Volcano plot of DESeq2 calculated changes in $\log_2$ fold-change in H3K27me3 signal in promoters versus the $\log_{10}$(p-value) in enrichment in JARID2Δ cells determined by CUT&RUN. CCND1 and CCND2 location within the dataset are indicated by yellow dots.
(S4B): Same as in (A) but for transcript abundance determined by RNA-seq.
(S4C): Scatter plot of $\log_2$ fold-change in transcript abundance vs H3K27me3 promoter signal for genes with an adjusted p-value <0.1 in our CUT&RUN and adjusted p-value <0.05 in our RNA-Seq from our JARID2Δ cell lines.
(S4D): Bedgraph of H3K27me3, transcript coverage and CpG island location within the CCND3 promoter region (chr6:41,940,179-4,195,017; CpG island coordinates: chr6:41,941,008-41,941,973).
(S4E): Quantification of protein signal from Western blot in Fig. 6G for CCND1 (*left*), CCND2 (*center*), and CCND3 (*right*) normalized to Actin. Each bar is the mean for three biological replicates, error bars ±StDev. *: p-value<0.05, **: p-value<0.005, ***: p-value<0.0005, n.s.: not significant, two tailed unpaired Student's t-test.
(S4F): Dot plot of $\log_2$ fold-change for indicated mRNAs in MTF2Δ and JARID2Δ cells as determined by DESeq2. Established cut of ±1 $\log_2$ fold-change in transcript abundance indicated by dashed grey line.
(S4G): Western blot for a panel of G1 regulators from whole-cell lysates of wild-type, SUZ12Δ, MTF2Δ and JARID2Δ cell lines from three independently isolated monoclonal knockouts. Membranes were probed with the indicated antibodies.

bioRxiv preprint doi: https://doi.org/10.1101/2024.03.18.585604; this version posted March 22, 2024. The copyright holder for this preprint (which was not certified by peer review) is the author/funder, who has granted bioRxiv a license to display the preprint in perpetuity. It is made available under aCC-BY 4.0 International license.

S1A

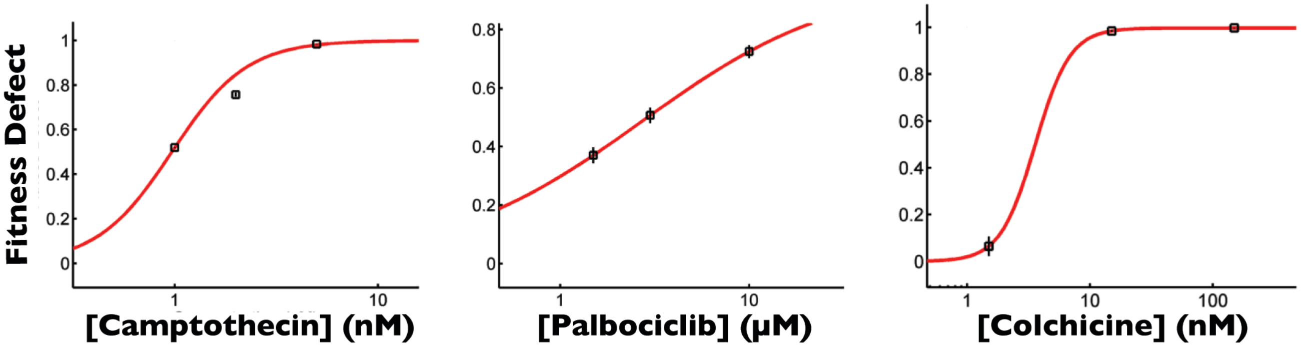

S1B

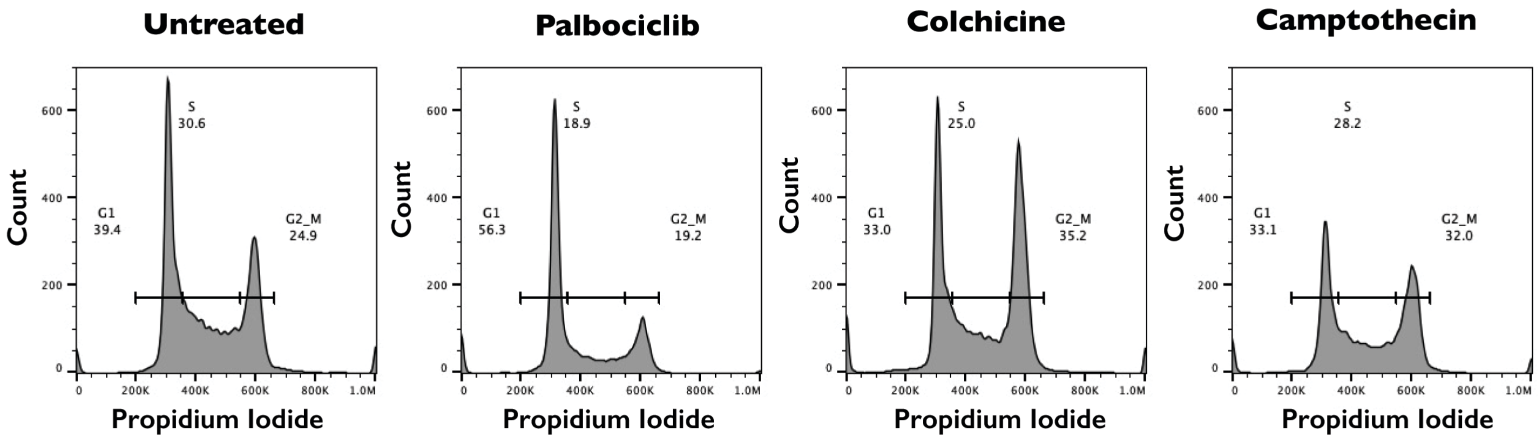

S1C

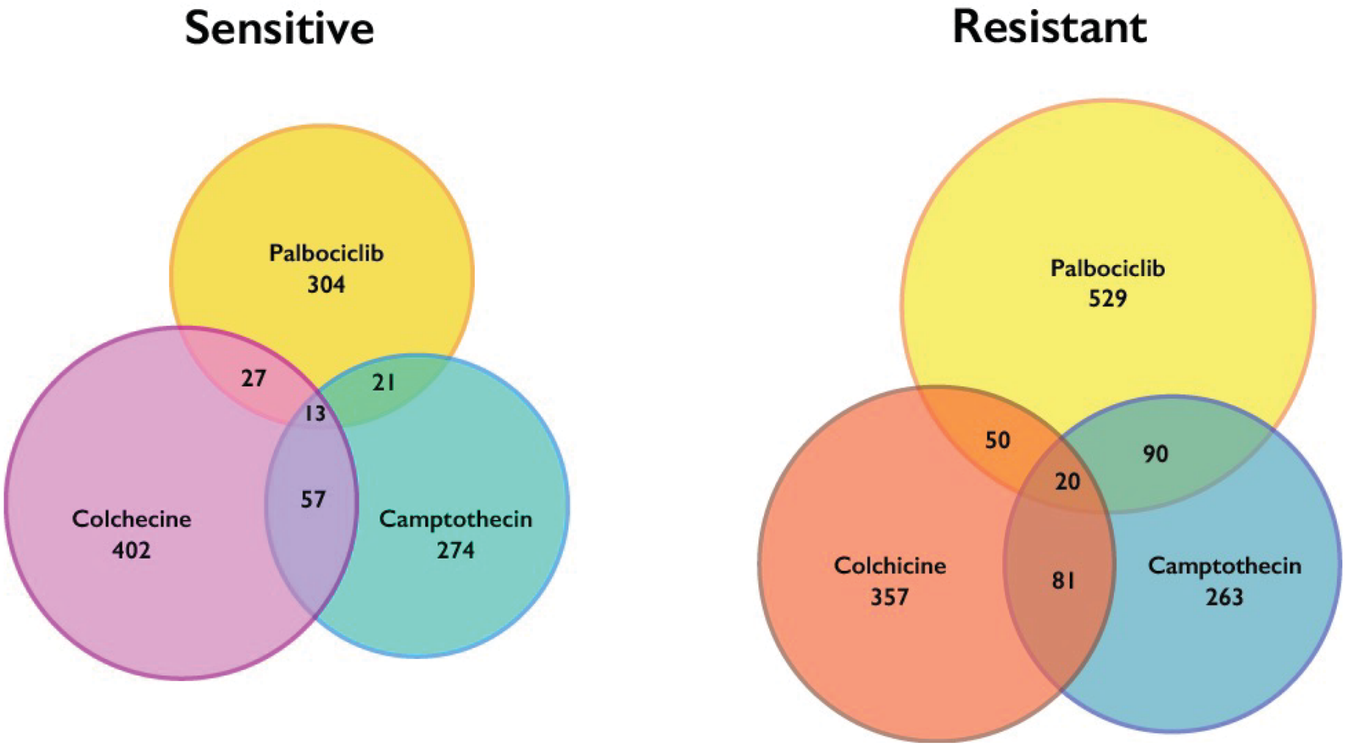

bioRxiv preprint doi: https://doi.org/10.1101/2024.03.18.585604; this version posted March 22, 2024. The copyright holder for this preprint (which was not certified by peer review) is the author/funder, who has granted bioRxiv a license to display the preprint in perpetuity. It is made available under aCC-BY 4.0 International license.

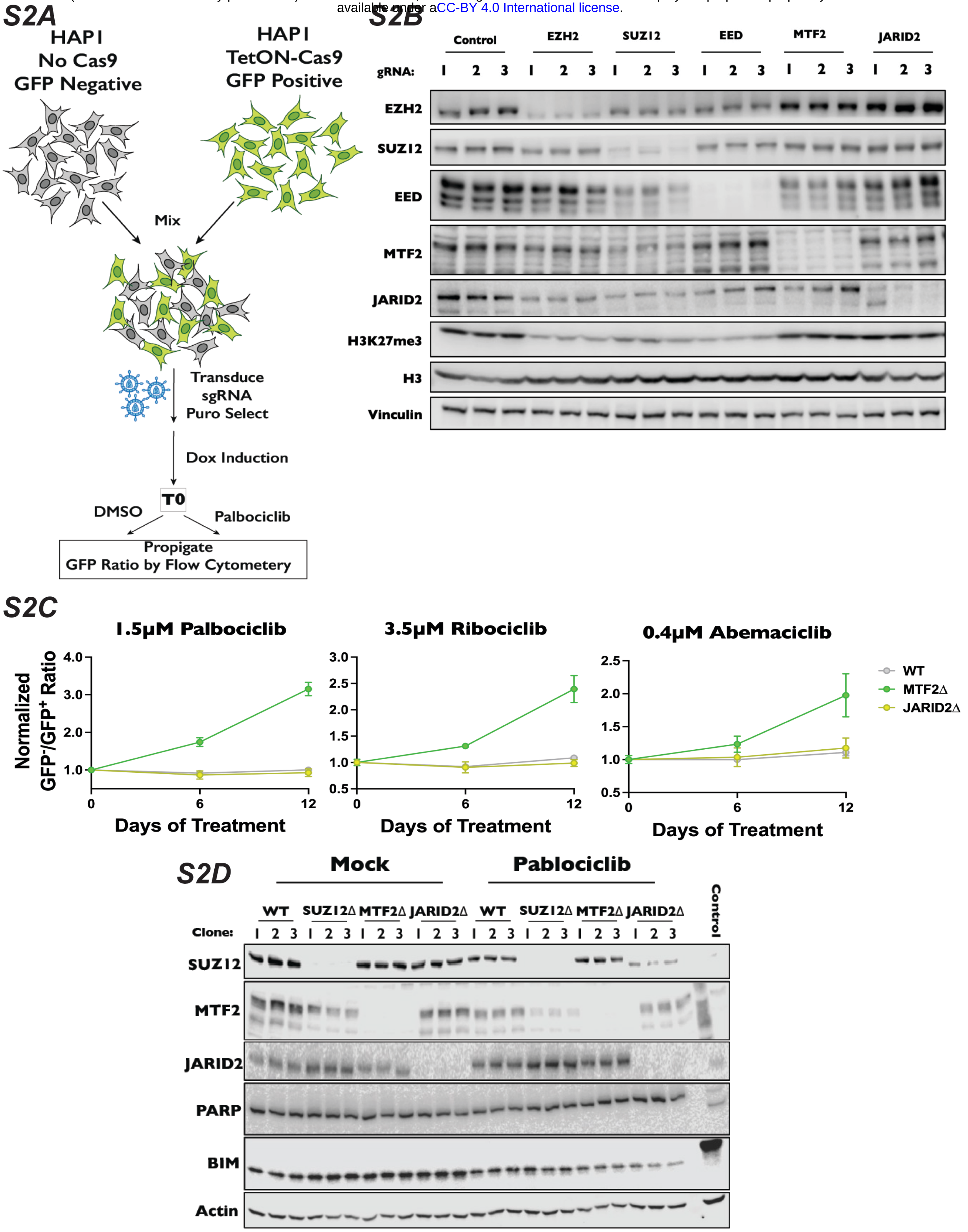

bioRxiv preprint doi: https://doi.org/10.1101/2024.03.18.585604; this version posted March 22, 2024. The copyright holder for this preprint (which was not certified by peer review) is the author/funder, who has granted bioRxiv a license to display the preprint in perpetuity. It is made available under aCC-BY 4.0 International license.

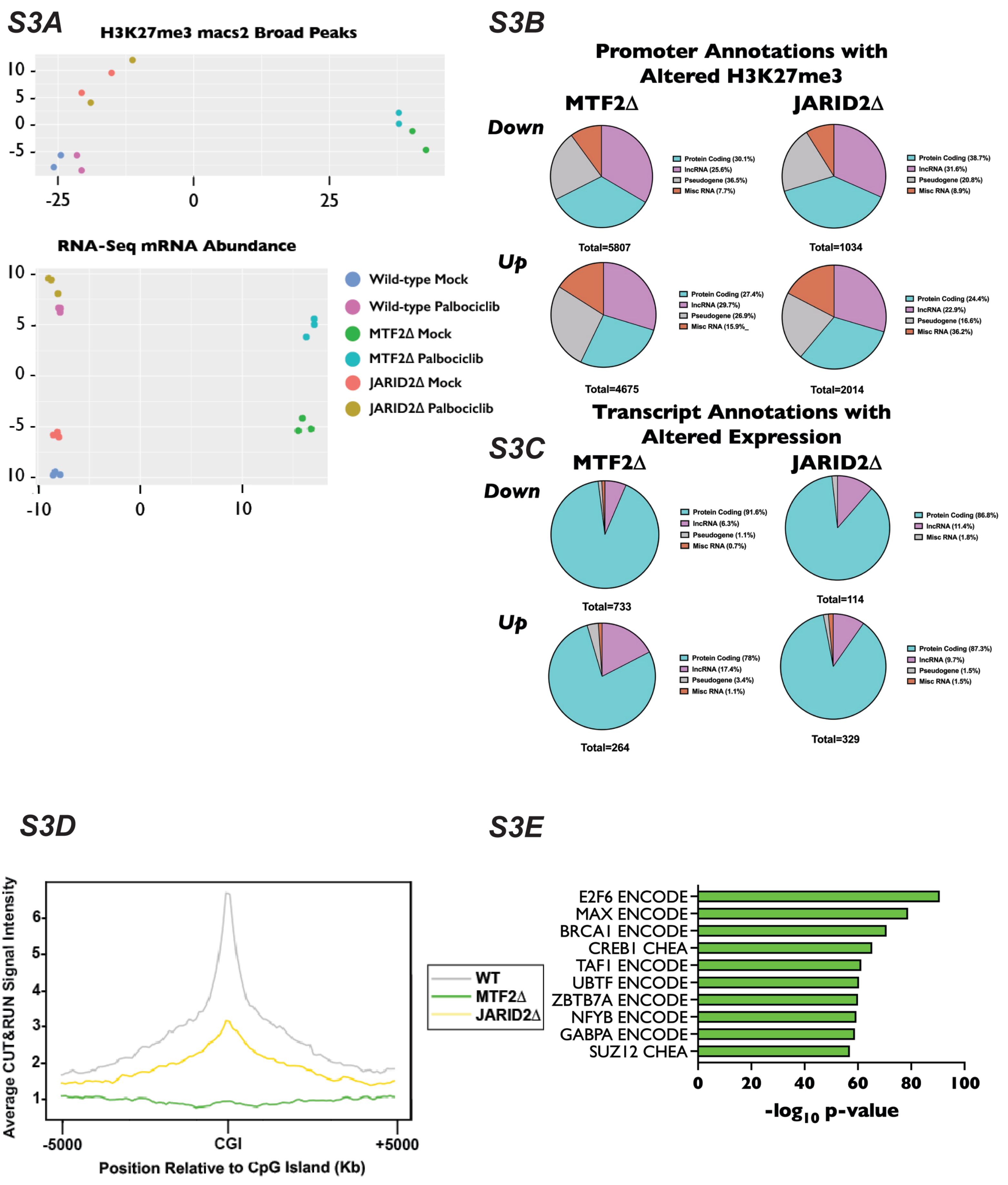

bioRxiv preprint doi: https://doi.org/10.1101/2024.03.18.585604; this version posted March 22, 2024. The copyright holder for this preprint (which was not certified by peer review) is the author/funder, who has granted bioRxiv a license to display the preprint in perpetuity. It is made available under aCC-BY 4.0 International license.

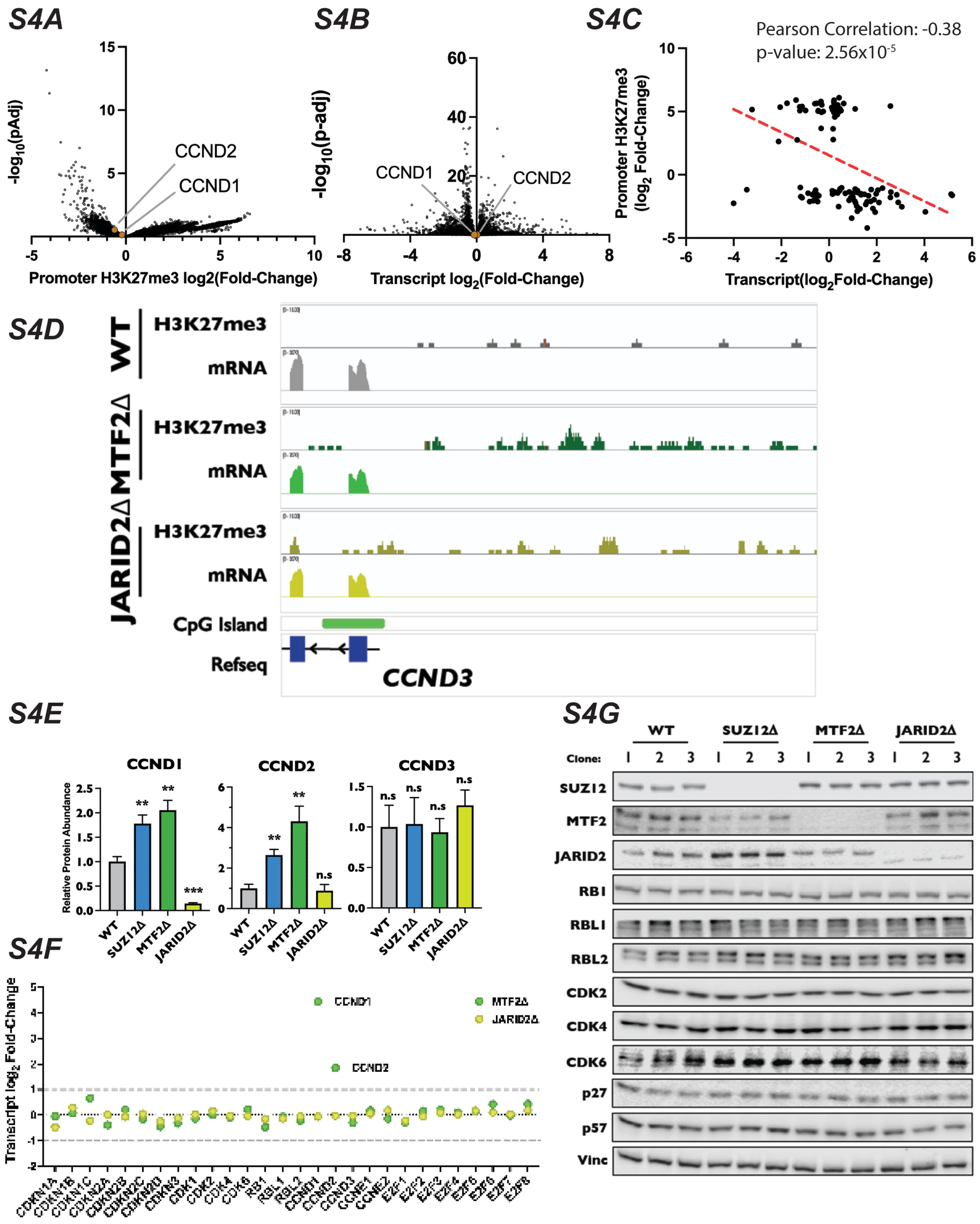

Supplement: Supplementary file 1 [file NIHPP2024.03.18.585604v1-supplement-1.pdf]
